# Supplementary material for: Traditional Chinese medicine Lianhua Qingwen treating corona virus disease 2019(COVID-19): Meta-analysis of randomized controlled trials
Source: PLoS One. 2020 Sep 11;15(9):e0238828. doi: 10.1371/journal.pone.0238828 (PMC7485773; doi:10.1371/journal.pone.0238828)
Supplement: S1 File — (DOC) [file pone.0238828.s002.doc]

JOURNAL REQUIREMENTS:

Contributions

Author：Linjun Li

Add： Writing – Original Draft Preparation

Modify the fund number：

Financial Disclosure: This work was supported by Sanya medical science and technology innovation project, No.: 2017YW06
