# Supplementary material for: Traditional Chinese medicine Lianhua Qingwen treating corona virus disease 2019(COVID-19): Meta-analysis of randomized controlled trials
Source: PLoS One. 2020 Sep 11;15(9):e0238828. doi: 10.1371/journal.pone.0238828 (PMC7485773; doi:10.1371/journal.pone.0238828)
Supplement: Attachment — Submitted filename: Response to Reviewers.doc [file pone.0238828.s003.doc]

Reviewer #1:

1. Is the manuscript technically sound, and do the data support the conclusions?

The application of meta-analysis avoids the limitations of a single small-sample clinical trial, makes the analysis results more comprehensive and reliable, and provides a good basis for medical decision-making. All data of Lianhua Qingwen's treatment of new coronary pneumonia are based on strict screening criteria to summarize and analyze published papers. However, the data of the selected two papers are based on clinical treatment. So the conclusion is reliable and reasonable.Thanks.

Reviewer #2:

2. Has the statistical analysis been performed appropriately and rigorously?

All data processing is carried out in accordance with strict standards, and it is also the conclusion that the three authors jointly discussed and decided to use special statistical tools.Thanks.

Reviewer #3:

3. Have the authors made all data underlying the findings in their manuscript fully available?

At that time, all the data were strictly screened and provided.Thanks.

Reviewer #4:

4. Is the manuscript presented in an intelligible fashion and written in standard English?

Without any plagiarism, we will be responsible for the originality of the manuscript. At the same time, I have consulted the editor of the magazine before. If necessary, you can forward the content of the email. At the same time, if there is plagiarism, our author will take the initiative to withdraw the manuscript immediately, and the reviewers are also asked to respect our results. All languages are carefully written and checked many times. If there are some problems, please point them out. Thanks.

Reviewer #5:

We have added some reasonable comments in the discussion section, and at the same time added a description that fits the article based on the actual situation of the manuscript.
